# Asymmetry of Edema Formation: The Possibility of a Somatic Mosaic

**DOI:** 10.7759/cureus.15335

**Published:** 2021-05-30

**Authors:** Michika Hamada, Hiroki Nagasawa, Ken-ichi Muramatsu, Kei Jitsuiki, Youichi Yanagawa

**Affiliations:** 1 Acute Critical Care Medicine, Juntendo University Shizuoka Hospital, Izunokuni, JPN

**Keywords:** allergy, somatic mosaic, edema, gynandromorphs, food

## Abstract

A 57-year-old woman experienced an abnormal feeling on the left side of her neck and difficulty breathing 90 minutes after eating Chinese noodles. She had a history of removal of a left sphenoid ridge meningioma one year earlier. She had experienced rigidity of her left neck and peripheral cold sensation on her left side in winter since approximately 10 years of age. She had experienced peripheral swelling of her left side and lower back pain of unknown origin on her left side several times. She had suffered for oral allergy syndrome since she was young. She sometimes experienced a tingling sensation on her lips and an unpleasant feeling in her throat after eating some types of fruit. On arrival, 180 minutes after eating the noodles, she had clear consciousness and stable vital signs. She had left neck and chest swelling without color change. Her difficulty breathing subsided spontaneously. A blood analysis revealed an increased level of immunoglobulin E. Neck computed tomography (CT) with contrast medium and magnetic resonance imaging (MRI) revealed left-side-limited edema in the subcutaneous area and surrounding esophagus and bronchus. These radiological findings denied hemorrhaging or pseudoaneurysmal formation. She underwent observational admission. After her edema improved, she was discharged on the third hospital day. A follow-up examination one week later showed the complete resolution of the neck and chest edema. A blood allergen test did not reveal the cause of the edema. The mechanism underlying the asymmetric transient edema after eating in the present case may involve somatic mosaic.

## Introduction

Mosaicism suggests that at least two genomes in an individual originated from a single zygote. Germline mosaicism is a mutation that is restricted to the gonads and which can be inherited by progeny. Somatic mosaicism is a postzygotic mutation that occurs in the soma, and it may occur at any formative stage or in mature structures [1]. As opposed to transmitted mutations, somatic mosaic mutations may influence solitary a segment of the body and are not inherited by progeny. These mutations influence shifting genomic sizes ranging from solitary nucleotides to whole chromosomes and have been involved in various illnesses, most remarkably, neoplasms.

The phenotype under somatic mosaicism is contingent on multiple components, including the formative time at which the mutation occurs, the segments of the body that are affected, and the pathophysiological effects of the mutation [2-5]. Gynandromorphs, which are congenitally masculine on one side of the centerline and genetically feminine on the other, represent the best model [6].

We herein report a case of transient asymmetric edema formation, possibly due to somatic mosaic.

## Case presentation

A 57-year-old Asian** **woman experienced an abnormal feeling on the left side of her neck and difficulty breathing 90 minutes after eating a Chinese noodle dish containing roasted pork, egg, spinach, seasoned bamboo shoots, laver, and garlic. She was advised to call an ambulance by our triage nurse after telephone consultation and was transported to our hospital by a physician-staffed helicopter due to the long distance from her house to the hospital. She had a history of removal of a left sphenoid ridge meningioma one year earlier. She had experienced limited rigidity of her left neck and limited peripheral cold sensation on her left side in winter since approximately 10 years of age. She had experienced limited peripheral swelling of her left side and limited lower back pain of unknown origin on her left side several times. She had been suffering from oral allergy syndrome since she was young. She sometimes experienced a tingling sensation on her lips and an unpleasant feeling in her throat after eating some types of fruit. Those fruits included mandarins, oranges, kiwis, bananas, and pineapples. She was married but did not have any children.

On arrival, 180 minutes after eating the noodles, she had clear consciousness, and her blood pressure was 130/90 mmHg, her heart rate was 80 beats per minute, her percutaneous oxygen saturation was 100% under room air, and her body temperature was 36.9°C. She had left neck and chest swelling without color change. Her breathing difficulty subsided spontaneously.

**Figure 1:**
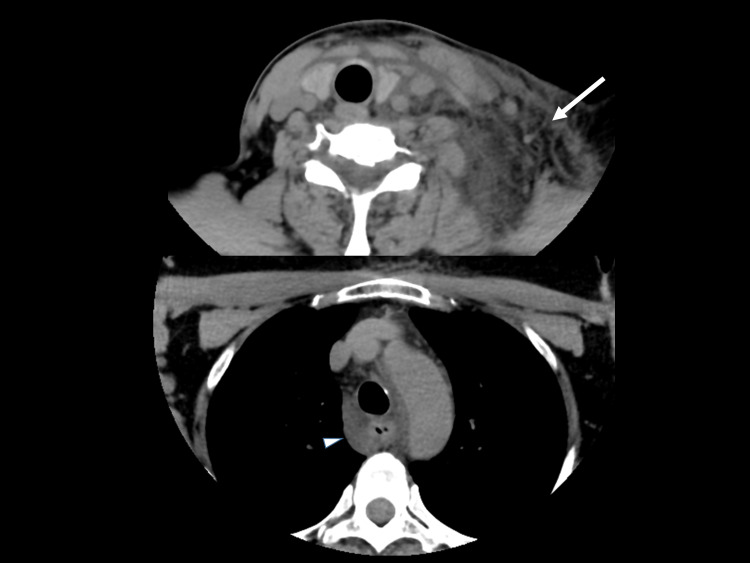
Trunk computed tomography (CT) on arrival CT showed left-side-limited edema (arrow) in the subcutaneous area and surrounding esophagus and bronchus (arrow head).

**Figure 2:**
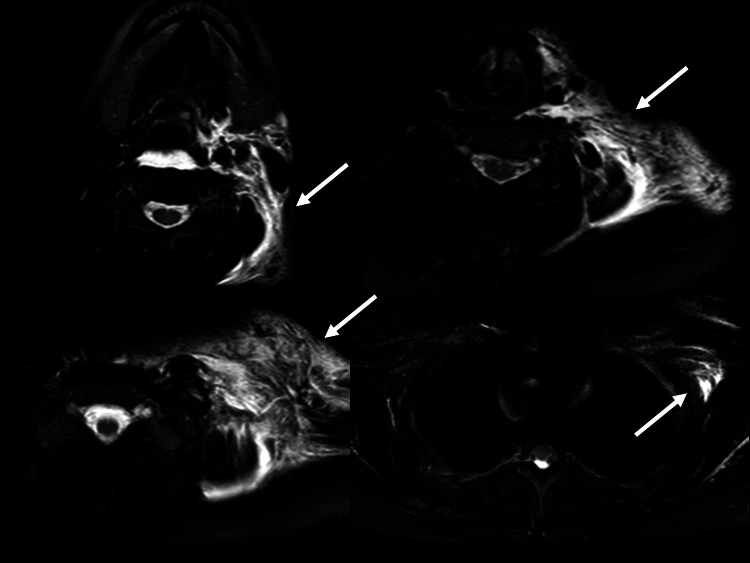
Neck magnetic resonance imaging on arrival Short T1 inversion recovery imaging showed left-side-limited edema (arrow).

The results of a blood analysis were as follows: white blood cells, 6,000/mm3; hemoglobin, 14.8 g/dL; platelets, 38.3 × 104/mm3; aspartate aminotransferase, 15 IU/L; alanine aminotransferase, 11 IU/L; total bilirubin, 0.5 mg/dL; total protein, 6.7 g/dL; glucose, 118 mg/dL; blood urea nitrogen, 18.9 mg/dL; creatinine, 0.90 mg/dL; amylase, 63 IU/L; creatine phosphokinase, 89 IU/L; sodium, 142 mEq/L; potassium, 4.0 mEq/L; chloride, 106 mEq/L; C-reactive protein, 0.05 mg/dL; prothrombin time, 10.6 (control, 11.9) sec; activated partial thrombin time, 26.4 (control, 26.9) sec; fibrinogen, 310 mg/dL; fibrin degradation products, 0.8 μg/mL; troponin T, 0.06 (0-0.14) ng/mL; and immunoglobulin E (IgE), 429 (0-250) IU/mL. Chest roentgen, an electrocardiogram and cardiac echography findings were all negative. Neck computed tomography (CT) with contrast medium and magnetic resonance imaging (MRI) revealed left-side-limited edema in the subcutaneous area and surrounding esophagus and bronchus (Figures 1, 2).

These radiological findings excluded hemorrhaging or pseudoaneurysm formation. She underwent observational admission. After her edema improved, she was discharged on the third hospital day. A follow-up examination one week later showed complete resolution of the neck and chest edema. The results of a blood allergen test analysis (0-1.39) showed an elevated immunoglobulin E (IgE) level as follows: Japanese cedar, 44.30; betula alba, 7.27; orchard grass, 3.42; Japanese cypress, 2.65; dermatophagoides farina, 3.03; house dust, 1.85; and mugwort, 1.70. The remaining allergens were within the normal ranges, as follows (0-1.39): alder 1.35, timothy 0.85, 0.33; cat skin, 0.25; and Candida, 0.22. The results for the following allergens were all zero: ragweed, Alternaria, dog skin, wheat, buckwheat, soybean, rice, tuna, salmon, lobster, crab, milk, beef, chicken, pork, albumen, ovomucoid, peanut, sesame, kiwi, banana and latex. We also obtained blood samples from the bilateral upper extremities at the same time. The examination items that showed ≥5% laterality were as follows: white blood cell count (6,500/mm3 vs. 6,100/mm3), platelets (40.8 × 104/mm3 vs. 38.0 × 104/mm3, aspartate aminotransferase (12 IU/L vs. 11 IU/L), lactate dehydrogenase (184 IU/L vs. 172 IU/L) and creatinine (0.63 mg/dL vs. 0.58 mg/dL). We could not obtain permission from the patient for further a genetic analysis by skin biopsy.

## Discussion

The transient neck and chest edema in the present case was thought to have been due to food allergy because the symptoms occurred after a meal, edema formed surrounding the upper esophagus, and there were increased levels of IgE for plants. A differential diagnosis other than food allergy may be hereditary angioedema because the present case had repeatedly experienced allergic reactions since she was young. Generally, the symptoms of hereditary angioedema have a slow, progressive onset and develop over several hours [7]. Untreated hereditary angioedema attacks tend to be more severe and persistent in comparison to a standard allergic reaction, typically persisting for 48-72 hours [7]. Furthermore, hereditary angioedema will not respond to standard treatments. However, the symptoms of the present case started soon after eating, were mild and subsided spontaneously [7]. Accordingly, the possibility of hereditary angioedema was considered to be minimal in the present case. The asymmetrical edema formation was attributed to somatic mosaic because the patient had had left-limited neck rigidity, peripheral cold sensation in the winter, repeated peripheral edema, and myofascitis, in addition to a left-sided brain tumor. Differences between the right and left body composition with regard to allergen sensitivity may be presumed to have resulted in the asymmetric edema formation in the present case. If true, the present case might be the first to show asymmetric edema formation induced by food allergy. This study is limited by the lack of a genetic analysis being performed between the right and left side of the body [8]. In addition, how to diagnose somatic mosaicism by a genetic analysis is a clinical question that should be addressed in the future, because the differences between the right and left, even after the analysis of all base sequences of the genome may simply be due to a mutation or indicate that a normal key gene produces asymmetry. To prove the gene causing mosaicism is difficult when the genes associated with normal asymmetry have not been specified. This possible explanation for the otherwise unexplained unilateral edema might be of interest to clinicians and heighten awareness about somatic mosaicism, even though there was not definitive attempt to prove this hypothesis.

## Conclusions

We presented a case of transient left-limited edema after eating. The mechanism underlying the asymmetric edema in this case may have been due to somatic mosaic.
